# The 2BFit study: is an unsupervised proprioceptive balance board training programme, given in addition to usual care, effective in preventing ankle sprain recurrences? Design of a Randomized Controlled Trial

**DOI:** 10.1186/1471-2474-9-71

**Published:** 2008-05-20

**Authors:** Maarten DW Hupperets, Evert ALM Verhagen, Willem van Mechelen

**Affiliations:** 1 EMGO Institute, Department of Public & Occupational Health, VU University Medical Center, Postbus 7057, 1007 MB, Amsterdam, The Netherlands

## Abstract

**Background:**

There is strong evidence that athletes have a twofold risk for re-injury after a previous ankle sprain, especially during the first year post-injury. These ankle sprain recurrences could result in disability and lead to chronic pain or instability in 20 to 50% of these cases. When looking at the high rate of ankle sprain recurrences and the associated chronic results, ankle sprain recurrence prevention is important.

**Objective:**

To evaluate the effect of a proprioceptive balance board training programme on ankle sprain recurrences, that was applied to individual athletes after rehabilitation and treatment by usual care.

**Methods/Design:**

This study was designed as a randomized controlled trial with a follow-up of one year. Healthy individuals between 12 and 70 years of age, who were actively participating in sports and who had sustained a lateral ankle sprain up to two months prior to inclusion, were eligible for inclusion in the study. The intervention programme was compared to usual care. The intervention programme consisted of an eight-week proprioceptive training, which started after finishing usual care and from the moment that sports participation was again possible. Outcomes were assessed at baseline and every month for 12 months. The primary outcome of this study was the incidence of recurrent ankle injuries in both groups within one year after the initial sprain. Secondary outcomes were severity and etiology of re-injury and medical care. Cost-effectiveness was evaluated from a societal perspective. A process evaluation was conducted for the intervention programme.

**Discussion:**

The 2BFit trial is the first randomized controlled trial to study the effect of a non-supervised home-based proprioceptive balance board training programme in addition to usual care, on the recurrence of ankle sprains in sports. Results of this study could possibly lead to changes in practical guidelines on the treatment of ankle sprains. Results will become available in 2009.

**Trial registration:**

ISTRCN34177180.

## Background

### Ankle injuries in sports

Ankle injuries are the most common injuries across a wide variety of sports [1-5]. It has been estimated that about 25% of all injuries across all sports are ankle injuries. Of all ankle injuries 85% involve the lateral ankle ligaments, i.e. acute lateral ankle sprains. The most recent count of sports injuries in the Netherlands (2000/2002) estimated that there was an absolute number of 1,200,000 acute sports injuries each year in a sporting population of 7,300,000 athletes [6]. A total of 120,000 ankle sprains were registered during this period, of which 43,000 (36%) required any form of medical treatment.

Recent research showed that the mean total costs (direct and indirect costs) of one ankle sprain are approximately € 360 [7]. This would give a rough estimate of the annual ankle sprains costs being € 43.200.000. Absence from paid or unpaid work was responsible for up to 80% of these costs [7]. In addition, there is strong evidence that athletes have a twofold risk for re-injury after a previous ankle sprain, especially during the first year post-injury [8-11]. These ankle sprain recurrences could result in disability and lead to chronic pain or instability in 20% to 50% of these cases [12].

The relatively high rate of ankle sprains across all sports, as well as the severity and subsequent negative consequences of ankle sprains on future sports participation motivates attention for preventive measures against this type of injury.

### Preventive measures

The most commonly used preventive measures against ankle sprains, i.e. tape and braces, have been evaluated in previous research. An overall ankle sprain reduction of approximately 50% can be attributed to any of these measures [13]. More recently a similar reduction in ankle sprain incidence has been ascribed to proprioceptive balance board training [14,15]. When comparing the preventive effects of tape, braces and proprioceptive balance board training a preventive effect was established only for players with previous ankle sprains. Players without a history of ankle sprains do not seem to benefit from these preventive measures. It is striking that this effect was established despite thorough treatment after an ankle sprain [14]. This suggests a secondary preventive effect, and could imply that the current rehabilitation after an ankle sprain is insufficient. For instance, the current guideline for acute ankle sprain by the Royal Dutch Physiotherapy Association (KNGF) states a rehabilitation period of no more than six weeks [16]. It is noted that after this period it could be decided individually whether further rehabilitation is needed. However, this prolonged treatment phase is intended specifically for high-level athletes and is aimed at reaching the personally desired loading capacity [16]. However, when looking at the high rate of ankle sprain recurrences and the known effectiveness of preventive measures, it is fair to state that prolonged rehabilitation after six weeks is needed in all athletes with ankle sprains in order to prevent ankle sprain recurrences.

Such a prolonged rehabilitation does not need to be supervised by a (sports) physician or (sports) physical therapist and might also suffice if the injured athletes are encouraged to follow a specific preventive programme for a certain period of time after their usual care. Such an unsupervised preventive programme would keep the medical costs associated with a prolonged rehabilitation period to a minimum, would put no additional demand on medical practitioners, and would have large potential positive effects in terms of health improvement and savings of direct medical costs due to ankle sprain recurrences. Another potential strength of an unsupervised programme lies in the fact that not all athletes with ankle sprains seek medical attention. However, the twofold-increased risk for an ankle sprain recurrence also exists in these athletes. Therefore, an unsupervised preventive programme targeted at ankle sprain recurrences would also benefit these athletes, and the unsupervised nature of such a programme makes it possible to administer the programme to these athletes through non-medical channels.

Proprioceptive balance board training is the most promising of the three mentioned preventive measures for such an unsupervised preventive programme [14]. Whereas taping and bracing are functionally supportive devices of the ankle, proprioceptive balance board training is aimed at changing ankle structural characteristics by re-strengthening muscles and ligaments and restoring proprioception of the damaged structures around the ankle [17-19]. Proprioceptive balance board training is an equally effective, easy to apply measure that is already widely used in the supervised rehabilitation after an ankle sprain [14,20]. Next to tape loosening, which was shown to occur after as little as ten minutes of exercise [21], tape can be irritating to the skin. Negative effects were shown for certain types of braces as well [20]. Moreover, the use of tape and/or braces is accompanied by relatively high costs [22], as compared to proprioceptive balance board training [23]. Costs of a proprioceptive balance board in this study was € 12.50, which is much lower compared to multiple roles of tape (costs: € 3.00 per roll), or a brace (costs: € 67.89 per brace) [24].

### Objective

The objective of this randomised controlled trial was to evaluate the effect of an unsupervised proprioceptive balance board training programme on ankle sprain recurrences, that was applied after the usual care of individual athletes who had sustained an acute sports-related lateral ankle ligament injury.

## Methods/Design

The CONSORT statement was followed to describe the design of this study [25,26]. This statement is a checklist intended to improve the quality of reports of randomized controlled trials.

### Study outline

**Figure 1:**
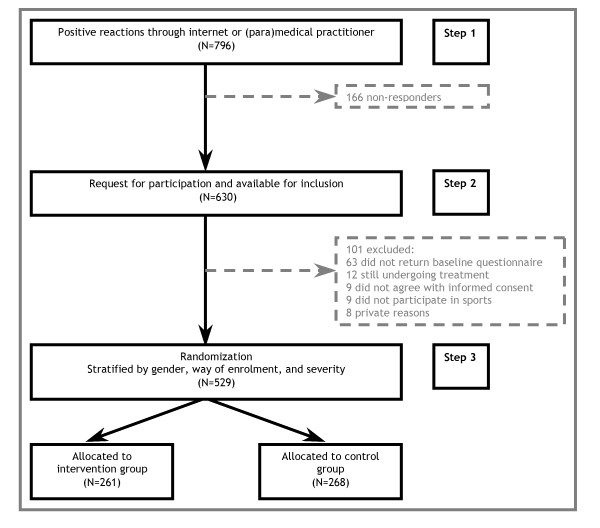
2BFit study flow chart.

The Balance Board Functional Instability Training (2BFit) study was a randomized controlled trial (RCT) with a one year follow-up. The study design and flow of participants are shown in Figure 1.

The focus of this study was to examine the effectiveness of an extended treatment procedure, on top of the standard usual care after an ankle sprain. Therefore, subjects in the intervention group received an eight week training programme following treatment by usual care, whereas the control group received usual care only.

The study was funded by the Netherlands Organization for Health Research and Development (ZonMW). The study design, procedures and informed consent procedure were approved by the Medical Ethics Committee (number 06/085) of the VU University Medical Center, The Netherlands. All participants provided written informed consent.

### Hypotheses

The first hypothesis of this study was that the intervention would lead to a 50% reduction of ankle sprain recurrence incidence. The second hypothesis was that the intervention would additionally lead to ankle sprain cost-reduction, mediated through a decrease of productivity loss and prevention of direct medical costs related to recurrent ankle sprains.

### Participants

Healthy participants between 12 and 70 years of age, who were actively participating in sports and who had sustained a lateral ankle sprain up to two months prior to inclusion, were eligible for inclusion in the study. Participants were excluded if they did not master the Dutch language, had a history of vestibular complaints, or in case of a diagnosis of a different injury than a lateral ankle sprain (e.g. fracture of the ankle).

This resulted in a diverse source population of athletes from all types and levels of sports were involved in the study. The intervention was considered to be appropriate for all athletes and to have no negative side-effects.

### Sample size

A power calculation was carried out for the main outcome variable ankle sprain recurrences. A difference of 50% in the incidence of recurrent ankle sprains between the intervention and control group after a follow up of 12 months was considered to be clinically relevant and was found previously in a volleyball setting [14].

The prevalence of ankle sprain recurrences being about 13% in 12 months [11], 275 subjects per group were needed to detect the intended difference of 50% in the incidence of acute ankle sprain recurrences, with a power of 80% and an alpha of 5%. Assuming a dropout rate of about 20% a total of 688 athletes were needed to detect a potentially clinically relevant effect of the intervention.

### Recruitment of study population

Recruitment was carried out at eleven hospital emergency rooms (ER's), five general practices, and four physical therapy offices throughout the Netherlands, assisted by advertisements in Dutch media (i.e. newspapers, sports magazines, sports tournaments and the internet). An information brochure was handed to individuals, participating in sports, who had sustained an ankle sprain and visited the cooperating (para-)medical institutes. If individuals were interested in participating in the study, they returned an answer form to the researchers. Enrolment through the internet was possible as well. The total number of positive reactions is shown as step 1 in Figure 1. Step 2 depicts the total number of individuals ready for inclusion. This phase started with contacting potential participants by phone. When contact was made, individuals were screened on their reported ankle injury and were asked if they were still interested to participate. Athletes who met the inclusion criteria were sent written information about the study along with an informed consent. The third step shows the total number of this convenience sample of individuals who were randomized to the intervention or control group. In this phase, a baseline questionnaire was sent after written informed consent was received. Stratified randomization was based on the completed baseline questionnaire.

Recruitment of participants for the 2BFit trial took place between August 2006 and August 2007. A total of 796 people were interested to participate in the 2BFit trial and reacted on the call for subjects with a recent ankle sprain. A total of 166 athletes were classified as non-responders after a maximum of ten attempts to contact them through telephone or email failed. Of 630 people who were available for inclusion, 101 were excluded. Reasons for exclusion were: no return of baseline questionnaire (63), still undergoing treatment at the end of inclusion period (12), no agreement on informed consent (9), not participating in sports (9), and private reasons (8). After baseline measurements and stratification, 529 athletes were randomly assigned to the intervention group (n = 261) and to the control group (n = 268).

### Randomisation procedure

After informed consent and baseline questionnaire, participants were randomized to the intervention training programme or the control group. To ensure that both groups were equal in terms of re-injury risk, a stratified randomization was performed (see table 1). Participants were stratified for gender, way of enrolment, and severity of their ankle sprain. Multiple studies have shown that women involved in similar activities as men are at increased risk for ligament injuries, such as ankle injuries [27] and ACL injuries [28-31]. The way of enrolment was used as a stratum to avoid selection bias. Registration through hospital ER's, through general practitioners, through physiotherapists, or through the internet were used as stratum options for the way of enrolment. Because hospital ER's had differing guidelines in treating ankle sprains, all eleven hospitals were judged as separate cells in this stratum. Utilisation of health care resources after an ankle sprain was judged as a measure of injury severity. There were four categories of medical treatment. The first category was no treatment needed after the athlete had suffered an ankle sprain or no further treatment after visiting a (para)medical practitioner. The second category was formed by paramedical treatment of the ankle sprain. Next to this option, the athlete utilized intramural medical treatment, or as a fourth category extramural medical treatment. From each stratum participants were allocated to the intervention and control group by random numbering.

**Table 1:** Strata used for subject randomization.

**Strata**	**Options**
Gender	Male
	Female
Way of enrolment	ER (11 strata)
	General practice
	Physical therapy office
	Worldwide web
Health care utilisation	No treatment
	Paramedical treatment
	Intramural medical treatment
	Extramural medical treatment

### Usual care of an ankle sprain in the Netherlands

There are three Dutch medical guidelines for the treatment of an ankle sprain. As stated in the introduction, in general, treatment duration of no more than six weeks is considered to be sufficient according to the KNGF (physiotherapy) guideline. High-level athletes could have a treatment duration of up to twelve weeks [16]. Therefore, the majority of amateur athletes will only have a rehabilitation period of six weeks. This guideline is aimed at optimal functional recovery of the ankle, at returning to full (or highest possible) sports participation, and at preventing recurrent ankle injuries [16]. The usual care treatment process starts with reducing pain and swelling, an improvement in blood circulation, and promoting partial loading. The next treatment phase consists of gradual increase of load and re-establishing function. After 11 to 21 days muscle strength can be improved, as well as active functional instability, range of motion and moving. The last phase aims to improve load and ADL tasks. The duration of each stage is prescribed in the guideline, but interindividual differences in treatment duration can occur.

The Dutch College of General Practitioners (NHG) practice guideline for ankle sprains states that follow-ups are not necessary for an individual with a mild sprain [32]. A mild sprain is diagnosed if the person had reasonably good ability to bear weight on the foot (walking), if there was mild swelling and pain, if there was no visible bruising, and if the anterior drawer test was negative. There should be improvement within one to two weeks. If not, the physical examination should be repeated. According to the NHG practice guideline, a person with a ruptured ligament is advised to be treated with a tape bandage and should be seen at two-week intervals for six-weeks [32]. After tape bandaging, patients who engage in sports with a high risk of inversion injury (such as football, basketball, outdoor hockey, volleyball) should be advised to use an ankle brace for secondary prevention [32].

The Dutch Institute for Healthcare Improvement (CBO) has formulated a consensus statement on diagnostics and treatment of acute ankle sprains [33]. This guideline is a multidisciplinary collaboration of several Dutch colleges of medical associations, of which the NHG is one. This guideline states that a maximum treatment duration of six weeks is sufficient [33]. It is noted that the advised treatment procedure consists of taping and bracing, whereas the use of proprioceptive balance board training as a treatment method is not discussed.

In this study the aim was to compare usual care of athletes with a recent lateral ankle sprain with a proprioceptive training programme after usual care. Usual care of the ankle sprain was by choice of the athlete; there was no interference from the authors.

### Description of the intervention

The intervention programme consisted of an eight-week proprioceptive training, which started after finishing the complete ankle sprain treatment procedure and from the moment that sports participation was again possible. From that moment athletes completed the baseline questionnaire and commenced their one-year follow-up.

**Figure 2:**
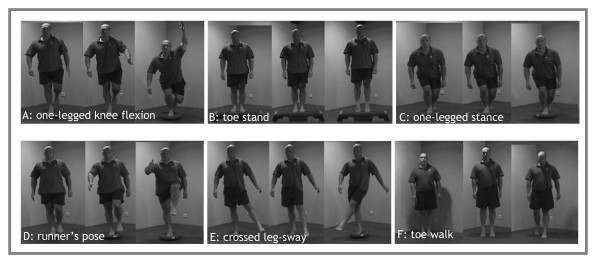
Basic exercises of the 2BFit proprioceptive balance board training programme.

The programme was a modification of the programme previously proven effective for the prevention of ankle sprain recurrences in volleyball [14]. The previously studied programme was designed specifically for volleyball (i.e. volleyball specific exercises) and had to be carried out in couples. The proprioceptive balance board training programme used for this study was similar to this previous programme, but consisted of more general exercises on and off the balance board that were carried out individually. The different basic exercises of the proprioceptive balance board training programme are depicted in Figure 2.

All participants of the intervention group received the same balance board, and general written and visual information on the duration and intensity of the programme. In addition, a website was created including basic information on the project and a section only accessible for intervention group subjects (including all exercises and a 'frequently asked questions' section).

The frequency of exercising was consistent throughout the full eight weeks: three training sessions per week (see Table 2). Participants were encouraged to perform the exercises as part of their warm up to their normal sporting activity. This was previously shown to be effective in a study on volleyball players [14]. Gradual increase of training load, that is, exercises became more difficult after several sessions (see Table 3).

**Table 2:** Eight week training programme for the intervention group.

	*Week 1*			*Week 2*			*Week 3*			*Week 4*			*Week 5*			*Week 6*			*Week 7*			*Week 8*		
	***1***	***2***	***3***	***4***	***5***	***6***	***7***	***8***	***9***	***10***	***11***	***12***	***13***	***14***	***15***	***16***	***17***	***18***	***19***	***20***	***21***	***22***	***23***	***24***
***A***	1	1	1	1	1	1	1	1	2	2	2	2	2	2	2	2	3	3	3	3	3	3	3	3
**B**	1	1	1	1	1	1	1	1	1	1	1	1	2	2	2	2	2	2	2	2	2	2	2	2
**C**	1	1	1	1	2	2	2	2	3	3	3	3	3	3	3	3	3	3	3	3	3	3	3	3
**D**	1	1	1	1	2	2	2	2	3	3	3	3	3	3	3	3	3	3	3	3	3	3	3	3
**E**	1	1	1	1	1	1	2	2	2	2	2	2	3	3	3	3	4	4	4	4	4	4	4	4
**F**	1	1	1	1	1	1	1	1	1	1	1	1	2	2	2	2	2	2	2	2	2	2	2	2

The content of each training session is shown vertically from session 1 to 24 (session numbers are printed bold). Exercises A through F are depicted as A to F. The numbers in all exercise rows represent the exercise difficulty level (see Table 2).

**Table 3:** Exercise difficulty levels

**Exc**	**Difficulty level**	**Exc**	**Difficulty level**
A	1. on even surface	E	1. on even surface; with handhold
	2. on even surface; eyes shut		2. on even surface; without handhold
	3. on balance board		3. on even surface; eyes shut and without handhold
B	1. on high surface; with handhold		4. on balance board
	2. on high surface; without handhold	F	1. on even surface; walking
C	Same 3 levels as exercise A		2. on even surface, jumping
D	Same 3 levels as exercise A		

All participants trained individually, without supervision of a coach or medical practitioner.

### Outcome measures

The primary outcome of the 2BFit study was the number of recurrent ankle injuries in both groups within one year after the initial sprain. A follow-up time of one year was chosen as a higher risk of re-injury was previously found during the first year post-injury [8-11]. An ankle sprain recurrence was recorded if the subject suffered a sudden inversion of the ankle, which caused the subject to stop his or her current sporting activity, or resulted in the participant not participating (fully) in the next planned sports or work activity.

Secondary outcome measures are:

1. Severity of re-injury

2. Etiology of re-injury

3. Medical care

This study had a one-year follow-up with measurements scheduled monthly for 12 months after baseline. All questionnaires and other forms were sent to athletes by mail, unless requested otherwise. The questionnaires were derived from validated questionnaires [34]. Where appropriate, questions were adapted to fit this particular study.

### Baseline measurement

The baseline questionnaire was the largest questionnaire and consisted of six parts. Part one covered demographic variables (i.e. gender, age, body weight, and body height), and type of sport. Sports participation was assessed in part two by means of questions concerning mean training hours per week, number of matches played per month, and participation in other sports. Part three covered the working situation including questions on mean working hours a week and type of occupation. For stratification purposes, part four consisted of a question about treatment after the initial diagnosis of ankle injury. Preventive measures were assessed in part five, with questions concerning use of braces, tape, and/or socks. Information about medical history was collected by the last part of the baseline questionnaire. Questions about previous ankle and knee injuries were assessed as well as other injuries of the lower extremities.

### Follow-up measurement

The monthly questionnaires gathered information on sports participation, on the use of preventive means and sustained knee or ankle injuries in the past month. Per training or match session the total minutes of participation, type of sports and participation percentage of total session duration were registered. In case of absence from a training or match session, the reason (injury, illness, motivation, or other reason) for it was asked.

### Compliance

During the 8 weeks proprioceptive training programme, the intervention group completed an additional questionnaire on the subjective response to the proprioceptive and balance board training programme, and reasons of (non-)compliance.

If participants did not fill in their questionnaires or forms after one week, a reminder was sent by email. If the questionnaires or forms were not returned after two weeks, contact was made by telephone.

### Injury registration

In case of an ankle sprain, athletes were asked to fill in a web-based ankle sprain registration form. This form consisted of questions concerning the diagnosis, the cause, and the etiology of the re-injury. Furthermore, the advised treatment and the person who treated the injury were registered. Based on this form, a cost diary was sent to the athlete.

### Cost diary

To evaluate the cost-effectiveness of the intervention programme subjects (intervention and control group) who sustained an ankle sprain received a cost-diary.

The cost-diary was a log, which registered all absence from work, school and other chores of life, and (para-)medical treatment (including use of medication) from the moment of injury onwards until full recovery. From these cost-diaries direct and indirect costs resulting from the sustained ankle sprain could be calculated for use in an economic evaluation.

### Cost-effectiveness evaluation

The economic evaluation was performed from a societal perspective. Table 4 provides an overview of the costs collected [35,36]. Cost of the intervention included costs that were directly related to the implementation of the intervention programme. These costs included the written information materials, an instructional video, development and maintenance of an informational website, and the balance boards. Besides the cost of the intervention itself, direct health care costs were included: i.e. costs of care by a general practitioner, physiotherapist, massage therapist, alternative therapist, and care by a sports physician or medical specialist (e.g., orthopaedic surgeon, general surgeon); hospital care; use of drugs (e.g., paracetamol/actaminophen, ibuprofen) and medical devices (e.g., crutches, tape, braces). The costs of drugs were estimated on the basis of prices recommended by the Royal Dutch Society of Pharmacy [37]. Also indirect costs resulting from a loss of production due to absenteeism from paid or unpaid work were included. Indirect costs for absenteeism from paid work were calculated using the friction cost approach of 4 months, based on the mean age and sex specific income of the Dutch population [24,36]. Indirect costs for productivity loss of unpaid work, such as study and household work, costs were estimated at a shadow price of € 7.94 an hour [35].

**Table 4:** Costs applied in the economic evaluation of a proprioceptive balance board training programme for the prevention of recurrent ankle sprains.

**Costs**	**Cost (€)**
*Direct health care costs*:	
General practitioner (per visit = 20 min)*	16.60
General practitioner (phone consult)*	8.17
Physical therapist (per visit = 30 min)*	18.15
Sports physician (per visit)*	16.60
Medical specialist (per visit)*	40.85
Alternative therapist^# ^(per visit)*	27.20
X-ray/cast^† ^(per unit)	50.00
Emergency room (per visit)^†^	50.00
Drugs^‡^	-
Medical devices^‡^	
Tape (per roll)	3.00
Brace	67.89
Crutches (rent per week)	15.00
*Indirect costs*:	
Absenteeism from paid work (per day)^§^	-
Absenteeism from unpaid work (per hour)*	7.94

€1.00 = €0.78, $1.56 (d.d. 04-03-2008)

* Guideline price according to Dutch guidelines [36].

# Price according to professional association.

† Cost price according to hospital administration of VU Medical Center

‡ Price according to tariff of the Royal Dutch Society of Pharmacy [24].

§Indirect costs for paid work was calculated for each injured separately based on mean income of the Dutch

population according to age and sex [36].

### Process evaluation

A process evaluation was conducted for the intervention programme. The supplement questionnaires, which were sent to intervention group participants in questionnaires two and three, contained questions on the subjective response to the programme, the expected effect of the intervention, and the compliance to the programme.

### Statistical analyses

To evaluate the success of the randomization, baseline values were analyzed for differences between intervention group and control group, using a chi-square for categorical data and a student's t-test for numerical data. Cox-regression analysis was used to compare ankle sprain recurrence risk between the intervention and control group. Other variables were checked for confounding and/or effect-modification and were adjusted accordingly. Severity of re-injury was tested between groups using a Mann-Whitney test, since it is shown to be not normally distributed [8,11,14,20]. Etiology and medical care were descriptive variables.

All analyses were carried out according to the 'intention to treat' principle. Differences were considered statistically significant at p < 0.05.

Mean direct, indirect and total costs were estimated and compared between the two groups, both for the costs per subject in the injured population and for the costs per subject in the total population. Because costs were not normally distributed, 95% confidence intervals for the differences in mean costs was obtained by bias corrected and accelerated bootstrapping (2000 replications) [38]. Differences in costs and differences in ankle sprain recurrences were included in a cost-effectiveness ratio, which estimated the additional costs to prevent one ankle sprain recurrence. Confidence intervals for the cost-effectiveness ratio were calculated with bootstrapping, using the bias-corrected percentile method with 5000 replications. Uncertainty regarding the estimate of this ratio was expressed on a cost-effectiveness plane.

## Discussion

The 2BFit trial is the first randomized controlled trial to study the effect of a non-supervised home-based proprioceptive balance board training programme in addition to usual care, on the recurrence of ankle sprains in sports.

### Recruitment

The recruitment of athlete participants in the 2BFit study was the most challenging task. Although a total of eleven participating hospitals was deemed sufficient to reach the desired number of participants within one year, the way of enrolment had to be adjusted. This total number of hospitals was based on the estimated annual number of acute ankle sprains in the Netherlands. The partaking hospitals had an estimated total of 4000 acute ankle sprains in 2003. A two-month pilot study was performed at one of the participating hospitals to identify the strong and weak points and feasibility aspects of the intended way of recruitment at the ER's. This pilot resulted in a high number of positive reactions from hospital employees as well as from athletes, and encouraged us to state that eleven hospitals was sufficient.

Nevertheless actual inclusion of participants through participating ER's was below expectations, namely 26 participants after two months, where 100 participants were expected. Therefore, it was chosen to approach general practitioners and physical therapists to participate in the project. Additionally, the internet was used as a mean to increase awareness of the existence of our programme. It was also possible to enroll through the webpage of the study.

In conclusion, although the recruitment of participants started of slow, appropriate measures were taken, and the number of responders was adequate at the end of the recruitment period.

### Co-intervention

It is striking that the only Dutch medical guideline explicitly describing the use of proprioceptive training for the treatment of acute ankle sprains is the KNGF guideline. This guideline states that proprioceptive balance board training could be useful as part of an ankle sprain treatment protocol [16]. It was possible that athletes randomized to the control group were subjected to proprioceptive training as part of their ankle sprain treatment by means of usual care. The aim of this study was to evaluate the added value of proprioceptive training on top of usual care, which made co-intervention of the proprioceptive balance board training programme impossible.

### Non-response

Between step 1 and step 2 of the study flow (see Figure 1) about twenty percent (n = 166) of a total number of 796 positive reactors were lost. This is a fairly high number of non-response after athletes declared their interest in participation. A possible reason for this high number of non-response could be that the way of contact was not appropriate. Possible participants had to be contacted through email or by phone, since that was the only information gathered from the answering forms. It turned out to be hard to reach athletes through email or by telephone. If athletes were contacted by postage mail, perhaps non-response would have been lower. Nevertheless, from an ethical point of view it was not allowed to have access to the patient's medical status. Another possible reason for the high number of non-response was that the ankle sprain is an easily forgotten injury; as soon as typical symptoms of an ankle sprain (i.e. pain, swelling) are over, athletes consider their ankle as healed.

### Loss to follow-up

Once athletes had given informed consent it turned out to be a struggle to retain them. As discussed, re-injury risk is highest in the first year after the initial ankle sprain [14]. Therefore athletes were prospectively followed for one year and were asked to fill in a web based questionnaire every month.

The compliance to the intervention programme was difficult to assess, since the proprioceptive balance board training was performed at home, without supervision. Athletes completed a compliance questionnaire each month during their training. Effort was made to make the training programme as appealing as possible: a parcel including a balance board, an instructional DVD, and instructional forms was sent to the athlete. Furthermore, athletes could log on to the internet and watch the training programme online.

### Impact of results

The results of this study could possibly lead to a change in the treatment of ankle sprains. Positive results on secondary outcomes could offer extended possibilities for implementation of the intervention in usual care. Positive study results could also lead to changes in the three practical guidelines on the treatment of ankle sprains. Results of this study will become available in 2009.

## Competing interests

The authors declare that they have no competing interests.

## Authors' contributions

EALMV conceived of the idea and obtained funding for the study. EALMV and MDWH developed the intervention, developed the design of this trial, and MDWH recruited participants. WvM provided advice on the study design and contributed to the content of the article. MDWH is the study investigator, was responsible for data acquisition, and wrote the article. All authors read and approved the final manuscript.

## Pre-publication history

The pre-publication history for this paper can be accessed here:


